# Small-molecule compound SYG-180-2-2 attenuates *Staphylococcus aureus* virulence by inhibiting hemolysin and staphyloxanthin production

**DOI:** 10.3389/fcimb.2022.1008289

**Published:** 2022-10-13

**Authors:** Lulin Rao, Yanlei Xu, Li Shen, Xinyi Wang, Huilin Zhao, Bingjie Wang, Jiao Zhang, Yanghua Xiao, Yinjuan Guo, Yaoguang Sheng, Lixia Cheng, Zengqiang Song, Fangyou Yu

**Affiliations:** ^1^ Department of Clinical Laboratory, Shanghai Pulmonary Hospital, School of Medicine, Tongji University, Shanghai, China; ^2^ School of Pharmaceutical Sciences, Wenzhou Medical University, Wenzhou, China; ^3^ Integrated Traditional Chinese and Western Medicine Hospital, Hangzhou, China

**Keywords:** *Staphylococcus aureus*, SYG-180-2-2, virulence, hemolysin, staphyloxanthin

## Abstract

Multi-drug resistant *Staphylococcus aureus* infection is still a serious threat to global health. Therefore, there is an urgent need to develop new antibacterial agents based on virulence factor therapy to overcome drug resistance. Previously, we synthesized SYG-180-2-2 (C_21_H_16_N_2_OSe), an effective small molecule compound against biofilm. The aim of this study was to investigate the anti-virulence efficacy of SYG-180-2-2 against *Staphylococcus aureus*. MIC results demonstrated no apparent antibacterial activity of the SYG-180-2-2. The growth curve assay showed that SYG-180-2-2 had nonlethal effect on *S. aureus*. Besides, SYG-180-2-2 strongly inhibited the hemolytic activity and staphyloxanthin synthesis in *S. aureus*. Inhibition of staphyloxanthin by SYG-180-2-2 enhanced the sensitivity of *S. aureus* to oxidants and human whole blood. In addition, SYG-180-2-2 significantly decreased the expression of *saeR*-mediated hemolytic gene *hlb* and staphyloxanthin-related *crtM*, *crtN* and *sigB* genes by quantitative polymerase chain reaction (qPCR). Meanwhile, the expression of oxidative stress-related genes *sodA*, *sodM* and *katA* also decreased. *Galleria Mellonella* assay revealed that SYG-180-2-2 was not toxic to larvae. Further, the larvae infection model showed that the virulence of bacteria was significantly reduced after 4 μg/mL SYG-180-2-2 treatment. SYG-180-2-2 also reduced skin abscess formation in mice by reducing bacterial burden and subcutaneous inflammation. In conclusion, SYG-180-2-2 might be a promising agent to attenuate the virulence of *S. aureus* by targeting genes associated with hemolytic activity and staphyloxanthin synthesis.

## Introduction

*Staphylococcus aureus* is one of the main pathogens causing hospital- and community-acquired infections, and can cause a range of infections, including skin and soft tissue infections, endocarditis, pneumonia and other life-threatening illnesses (Blot et al., 1998; Tong et al., 2015). With the use of various antibacterial drugs, the antimicrobial resistance of *S. aureus* has increased. In particular, the emergence of methicillin-resistant *S. aureus* (MRSA) has burdened the public health. Some reports showed that the prevalence of hospital-acquired MRSA had reached 50.4% in China and that of the community-acquired MRSA had reached 46.1% in Peru (Shang et al., 2016; Cabrejos-Hirashima et al., 2021). *S. aureus* can also produce a series of toxins, such as hemolysin, enterotoxin and exfoliative toxins (Oliveira et al., 2018).

The hemolysins produced by *S. aureus* include α-hemolysin, β- hemolysin, γ- hemolysin and δ- hemolysin. β- hemolysin is one of the pore-forming toxins, which is a 39 kDa protein containing 330 amino acids and can produce toxic effects on cells such as polymorphonuclear leukocytes, monocytes and T lymphocytes (Projan et al., 1989; Walev et al., 1996). In *S. aureus*, the SaeRS two-component system (TCS) plays an important role in controlling the production of various virulence factors (Liu et al., 2016). The *sae* system encodes four genes (*saeP*, *saeQ*, *saeR* and *saeS*) and affects the transcription of *hla*, *hlb* and *coa* (Mainiero et al., 2010). Gao et al. (Gao et al., 2017) showed that the golden carotenoid pigment is also one of the important factors affecting the virulence of *S. aureus*. The pigment protects *S. aureus* from host oxidant killing and enhances its toxicity in a subcutaneous abscess model (Liu et al., 2005). In *S. aureus*, there are five genes related to staphyloxanthin biosynthesis, including *crtM*, *crtN*, *crtP*, *crtQ*, and *crtO*, which are organized by the *crtOPQMN* operon (Pelz et al., 2005). SigB plays an important role in the regulation of the *crtOPQMN* operon, thereby affecting the synthesis of staphyloxanthin (Xue et al., 2019). Therefore, the development of agents targeting hemolysin and staphyloxanthin production is also a potential way to treat *S. aureus* infection.

SYG-180-2-2(C_21_H_16_N_2_OSe), a small molecule compound, contains an indole ring, a selenyl group, and an amido group. Indole are widely used as a privileged scaffold for the design of medicinal drugs (Huffman and Padgett, 2005; Higuchi and Kawasaki, 2007; Liu et al., 2009; Biersack and Schobert, 2012), and 3-selenylindoles have been identified as an important class of indole compounds with biological activity (Nogueira et al., 2004). Amido group, as a crucial substituent in pharmaceutical chemistry, are widely found in natural products and drug candidates, and exhibit a wide range of biological activities including anti-tumor and antiviral (Fatahala et al., 2017; Garg et al., 2019). In our knowledge, there is also no study on novel anti-virulence drugs combined with 3-selenylindole and the amido group. Following our discovery of anti-biofilm ability of SYG-180-2-2 (Rao et al., 2021), we are interested in exploring the anti-virulence of this new compound.

In the present study, we chose two *S. aureus* strains named SA75 and Newman to investigate the effect of SYG-180-2-2 on the virulence of *S. aureus*. The aim of this study was to demonstrate that SYG-180-2-2 acts by inhibiting the pigment synthesis and hemolysin release of *S. aureus*.

## Materials and methods

### Bacterial strains, reagents and culture conditions

The bacterial strains used in the study were SA75 and Newman. SA75 is a clinical *S. aureus* isolated from a patient with skin suppurative infection, which had remarkable hemolytic activity and pigment formation. The *S. aureus* ATCC 25904 Newman has the same phenotypic characteristics as SA75. All strains were incubated in trypticase soy broth (TSB) medium at 37 °C with shaking at 220 rpm. SYG-180-2-2 was synthesized by the School of Pharmacy, Wenzhou Medical University (Sheng et al., 2021). The details of the molecular structure, synthesis process and characterization of SYG-180-2-2 were shown in our previous study (Rao et al., 2021).

### Determination of MIC and growth assay

SYG-180-2-2 was prepared in dimethyl sulfoxide (DMSO, Biosharp, Beijing, China) at a concentration of 20 mg/mL. The broth microdilution method based on CLSI guidelines was used to determine the minimal inhibitory concentration (MIC) (CLSI, 2019). Specific methods for MIC values and growth curves of SYG-180-2-2 against *S. aureus* strains were described in our previous study (Rao et al., 2021).

### Hemolysis assay

The hemolytic activity of strains treated with SYG-180-2-2 was measured using sterile defibrillation rabbit blood. *S. aureus* strains were grown in TSB with or without 4 μg/mL SYG-180-2-2. After 16 h of incubation, cultures were adjusted to the same optical density (OD_600_) and then centrifuged at 8000 rpm for 5 min at room temperature. Next, 200 µL of supernatant was added to 800 µL of phosphate-buffered saline (PBS) with a final concentration of 2.5% sterile defibrillation rabbit blood. Blood with Triton X-100 and PBS were used as positive (100% hemolysis) and negative (0% hemolysis) controls, respectively. Subsequently, the samples were incubated at 37°C for 1 h. Then, the mixtures were centrifuged at 8,000 rpm for 5 min and the absorbance of supernatants was measured at 600 nm. Hemolysis (%) = [(absorbance of the treated sample – absorbance of negative control)/(absorbance of positive control – absorbance of the negative control)] × 100%. All assays were performed in triplicate.

### Quantitative enzyme-linked immunosorbent assay for alpha-hemolysin

The *S. aureus* SA75 and Newman were cultured in TSB with or without SYG-180-2-2 (4 µg/mL) for 24 h and cultures were normalized to a same optical density at OD_600_. Then, the supernatant was collected and filtered with a 0.22-µm filter. The α-toxin level was quantified by Staphylococcal α-Toxin ELISA kit (Chenglin Biological Technology Co., LTD, Beijing, China) following the manufacturer’s instructions. The test was performed independently in triplicate.

### Measurement of pigment production

The measurement of pigment production was assessed as previously described with some modifications (Chen et al., 2016). In brief, the *S. aureus* SA75 and Newman were cultured in 4 mL of TSB medium with or without SYG-180-2-2 at 37°C for 48 h, with shaking at 220 r.p.m. 3 mL of bacteria cultures were centrifuged, washed twice with PBS and then resuspended in 1 mL of methanol for extraction of the pigment. Subsequently, the absorbance was measured at 450 nm for determination of the pigment concentration. Pigment inhibition (%) = [(OD_450_ of control sample – OD_450_ of the treated sample)/OD_450_ of control sample] × 100%. The experiments were done in triplicate.

### Hydrogen peroxide killing assay

SA75 and Newman were grown in TSB with or without SYG-180-2-2 (4 µg/mL). After 2 days, the bacteria were washed twice with PBS and diluted to a concentration of 1 × 10^7^ CFU/mL. 250 μL of bacterial suspension was added in a 2-mL Eppendorf tube. Then, hydrogen peroxide (H_2_O_2_) was added to a 1 mM final concentration and the tubes were incubated at 37°C for 1 h with shaking at 220 r.p.m. The reaction was stopped by the addition of 1,000 U/mL of exogenous catalase (Sigma-Aldrich). Then, the cells were serially diluted with PBS and spread on the TSA plates. After incubating at 37°C for 24 h, viable cells were counted to assess whether SYG-180-2-2 affected the sensitivity of *S. aureus* to H_2_O_2_. All tests were run in triplicate.

### Human whole-blood killing assay

SA75 and Newman were grown in TSB with or without SYG-180-2-2. Overnight cultures were centrifuged at 1,2000 rpm for 1 min at room temperature and adjusted to a concentration of 1 × 10^7^ CFU/mL using sterile PBS. The bacterial suspensions were mixed gently with whole blood collected from healthy human volunteer at a ratio of 1:4 in 2-mL Eppendorf tubes (500μL). The tubes were incubated at 37°C for 6 h with shaking (220 r.p.m.) and bacterial viability was determined by plating dilutions on TSA plates. All experiments were run in triplicate.

### RNA isolation and RT-qPCR

*S. aureus* strains were cultured in TSB medium treated with either SYG-180-2-2 at the concentration of 4 μg/mL or without drug, and incubated at 37°C at 220 rpm. After 16 h, bacterial cells were centrifuged at 1,2000 rpm for 1 min at room temperature, resuspended in 80 μL of 20 mg/mL lysozyme and 4 μL of 1 mg/mL lysostaphin and incubated at 37°C for 1 h. Total RNA was isolated and quantified by a Nano-drop instrument (Thermo Fisher).

Then, total RNA (1 μg) was reverse transcribed into cDNA and the qPCR was performed. The primer pairs used in qPCR experiment are listed in **Table 1**. Specific kits, instruments and parameters for RT-qPCR are shown in our previous studies (Rao et al., 2021). The *gyrB* gene was used as an internal reference to normalize the expressions of genes of interest and PCRs were performed in 20 μL reaction mixtures. The relative quantification method (2^-ΔΔCt^) was used to analyze the transcription level of target genes. All analyses were conducted in triplicate.

**Table 1:** List of primers used in this study.

Primer	Sequence (5’-3’)
*gyrB*-RT-F	ACATTACAGCAGCGTATTAG
*gyrB*-RT-R	CTCATAGTGATAGGAGTCTTCT
*saeR*-RT-F	GTCGTAACCATTAACTTCTG
*saeR*-RT-R	ATCGTGGATGATGAACAA
*saeS*-RT-F	CGTTCTTGTAGTTCTGGTAT
*saeS*-RT-R	GTTGGTAGTCGCATTGATA
*hla*-RT-F	CTCGTTCGTATATTACATCTAT
*hla*-RT-R	GGTATATGGCAATCAACTT
*hlb*-RT-F	CGTAGCGATTGTAAGTAA
*hlb*-RT-R	TCTTCAGATTGTGTATGTG
*crtM*-RT-F	CACTTCAGCAATATCAACT
*crtM*-RT-R	AACACATCAGACATACGA
*crtN*-RT-F	AATGCTGAACAAGAGTAATC
*crtN*-RT-R	AGTGAATGGTGACATAAGA
*sodA*-RT-F	CCTCAGTTAATGGATTATCTTGGT
*sodA*-RT-R	TGGGCTTGGTTAGTCGTAA
*sodM*-RT-F	AGAGTTAGAGCATCAATCAC
*sodM*-RT-R	CCATTATTACGGACTGACAT
*katA*-RT-F	GATGGATACGGCTATGAAT
*katA*-RT-R	TGTAACAATGACGAATATGAC
*sigB*-RT-F	TTCCATTGCTTCTAACACTT
*sigB*-RT-R	GATGAACTAACCGCTGAAT

### Toxicity of SYG-180-2-2 to larvae of *galleria mellonella*

The toxicities of the SYG-180-2-2 was tested by inoculating doses of 0.32 mg/kg to groups of 10 larvae. This therapeutic dose exceeded the dose used in this study. Sterile PBS was inoculated as a negative control. The larval mortality of the treatment group and the control group was observed for three consecutive days.

### Larvae infection model

*S. aureus* strains were cultured in TSB with or without 4 μg/mL SYG-180-2-2 overnight at 37°C with shaking. Then bacteria were centrifuged at 1,2000 rpm for 1 min at room temperature, washed twice with PBS, and resuspended to 1 MacFarland standard (3.0 × 10^8^ CFU) in PBS. Groups of 10 larvae were inoculated with 10 μL of suspension, *via* last left forelimb using Hamilton syringe. In addition, one group of larvae was injected sterile PBS as a negative control. After the injection, each group of larvae was placed in a clean petri dish at 37°C. The mortality of larvae was observed every 1h for 12 times and then every 12 h to 3 days. The experiment was repeated more than three times.

### The safety of SYG-180-2-2 to the skin of mouse

Six-week-old female BALB/C mice were purchased from Shanghai SLAC Laboratory Animal Co., Ltd. (Shanghai, China). At the time of the experiment, they weighed approximately 20 ± 2 g. Briefly, the fur on the backs of mice was removed using depilatory cream (Reckitt Benckiser Plc.). There were two groups of eight mice each. The safety of the SYG-180-2-2 was tested by injecting 100 μL of 8 μg/mL SYG-180-2-2 on the skin of mice every 24 h for 3 days. Sterile saline was injected as a negative control. On the fourth day, the skins of the mice were observed and the skin of one mouse in each group was taken for pathological section.

### Mouse model of skin abscess infection

The *S. aureus* strains were cultured for 3 h at 37 °C, and then the bacteria were harvested and washed twice with sterile PBS. The number of bacteria was determined by reading the literature and pre-experiments before the mice were infected (Chen et al., 2016; Haney et al., 2018). For each experimental group, seven mice were used. Mice were inoculated with 100 μL of 7.5 × 10^6^ CFU of *S. aureus* SA75 or 100 μL of 1.5 × 10^7^ CFU of *S. aureus* Newman subcutaneously, and at 1 h post-infection, saline containing equal concentration of DMSO (for controls) or 4 μg/mL SYG-180-2-2 was given directly into the subcutaneous space of the infected area every 24 h for 3 days. The area of skin abscesses was recorded daily using a caliper. The length (L) and width (W) of abscess were measured and calculated as follows: A =π (L×W)/2. All animals were killed 4 d after infection. Skin abscesses were excised and homogenized by an automatic tissue homogenizer. There were seven mice in each group, the skin abscesses of six mice were used for viable cell counting and one mouse skin abscess for histopathological analyses.

### Statistical analysis

All data was analyzed using GraphPad Prism (version 8.0) and presented as mean ± SD (standard deviation). Two-tailed *t*-tests were performed for the experiments in this study. The difference was considered to be statistically significant when a *p* value less than 0.05.

## Results

### SYG-180-2-2 has no effect on the growth of *s. aureus* strains

**Figure 1:**
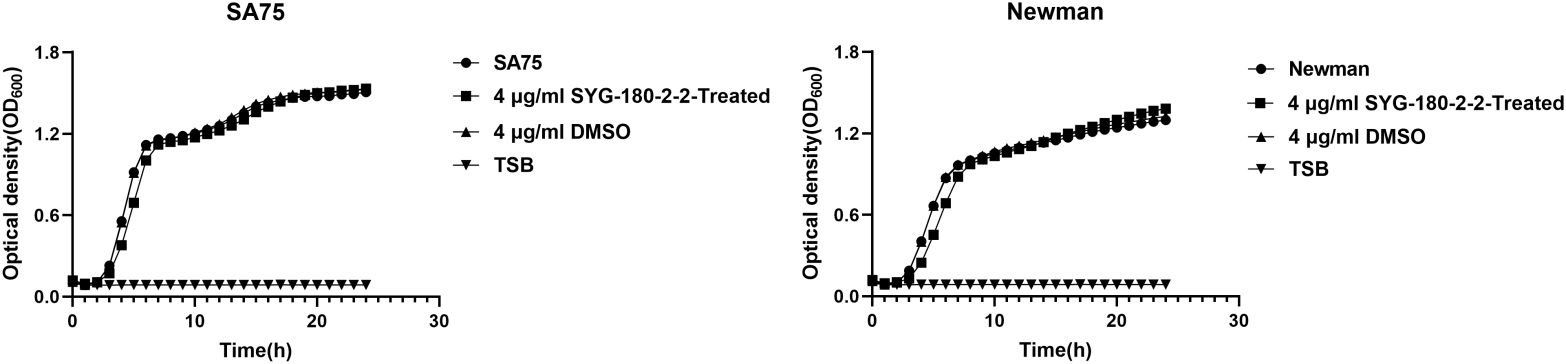
Growth curves of SA75 and Newman strains cultured with 4 μg/mL or without SYG-180-2-2. TSB was used as a blank control.

The MIC values of SYG-180-2-2 against SA75 and Newman were > 128 μg/mL. At 4 μg/mL of SYG-180-2-2, the OD of bacteria at the late logarithmic growth period was consistent (**Figure 1**). Our prior growth curve experimental data showed that SYG-180-2-2 reduced amounts of JP5023 and JP4856 growing at 8 μg/mL (Rao et al., 2021). However, SYG-180-2-2 did not reduce the number of SA75 and Newman at 8 μg/mL (**Supplementary Figure 1**). Here, we choose a lower concentration of 4 μg/mL to conduct the following studies.

### SYG-180-2-2 inhibits the hemolytic activity of *s. aureus*, while not *via* alpha-hemolysin

**Figure 2:**
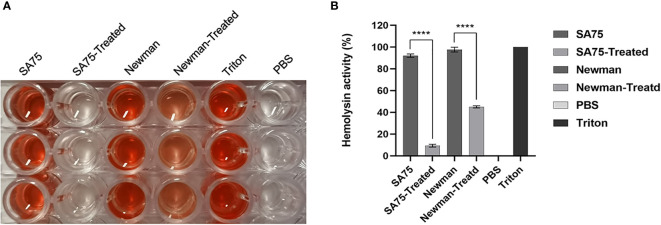
Effect of SYG-180-2-2 on the hemolytic activity of *S. aureus* strains. Triton X-100 and PBS were used as positive and negative control, respectively. **(A)** The images show the hemolysis of supernatant. **(B)** The percentage of hemolysis. ^****^ *P* < 0.0001.

The effect of SYG-180-2-2 on hemolysis activity of *S. aureus* supernatants was determined by comparing the hemolysis percentage of treated and untreated groups. As shown in **Figure 2A**, the hemolysis activity of the untreated group was significantly higher than that of the treated with SYG-180-2-2. For SA75, the hemolysis rate of without SYG-180-2-2 treated group was 92.53 ± 1.32%, and after being treated, its rate decreased by 80.70 - 85.97%. For Newman, the hemolysis rate of without SYG-180-2-2 treated group was 97.80 ± 2.19%, and after being treated, its rate decreased by 50.00 - 56.14% (**Figure 2B**).

Then, an ELISA kit was used to quantitatively detect the level of alpha-hemolysin between untreated strains and treated strains to explore whether SYG-180-2-2 could reduce its hemolysis activity by reducing α-toxin. For SA75, the α-hemolysin production of the untreated group was 28.84-37.63 pg/mL, and after being treated with SYG-180-2-2, its production was 27.87-33.40 pg/mL. For Newman, the α-hemolysin production of the untreated group was 31.77-35.35 pg/mL, and after being treated with SYG-180-2-2, its production was 32.10-35.03 pg/mL. These results showed that there was no significance difference between the treated and untreated groups (**Supplementary Figure 2**).

### SYG-180-2-2 significantly prevents the production of staphyloxanthin

**Figure 3:**
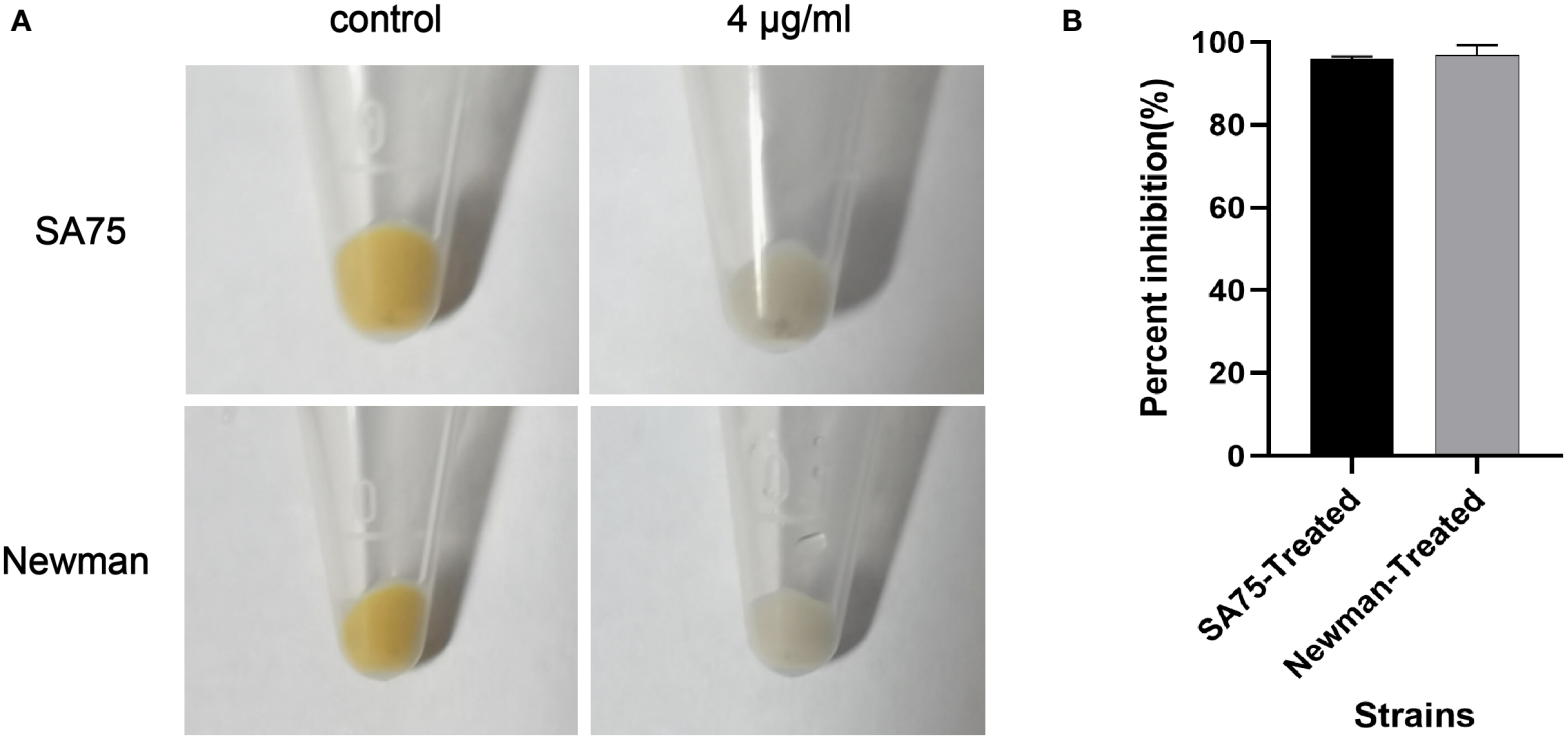
Effect of SYG-180-2-2 on wild-type *S. aureus* SA75 and Newman pigmentation. **(A)** The images show the spun-down cells. **(B)** Inhibition rate of pigment formation.

SYG-180-2-2 has potent activity against *S. aureus* staphyloxanthin formation *in vitro* according to the qualitative and quantitative experiments of staphyloxanthin synthesis. As shown in **Figure 3A**, the staphyloxanthin formation of the untreated group was yellowish, while that of the treated with SYG-180-2-2 group was colorless. After being treated with SYG-180-2-2, staphyloxanthin inhibition rates of SA75 and Newman were 96.04 ± 0.57% and 96.26 ± 2.31%, respectively (**Figure 3B**).

### SYG-180-2-2 sensitizes *s. aureus* to human whole blood and H_2_O_2_

**Figure 4:**
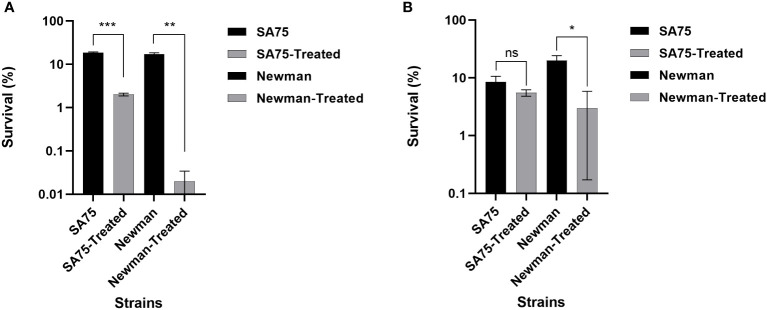
Effect of SYG-180-2-2 on the susceptibility of *S. aureus* SA75 and Newman to killing by either H_2_O_2_ **(A)** or human whole blood **(B)**. ^*^ *P* < 0.05, ^**^ *P* < 0.01 and ^***^ *P* < 0.001 and ns: not significant.

Because SYG-180-2-2 inhibited the production of *S. aureus* pigment (**Figure 3A**), and once the pigment was blocked, it may increase the sensitivity of bacteria to oxidative killing, so we compared the sensitivity of SYG-180-2-2-treated and untreated *S. aureus* to H_2_O_2_. Survival of the SYG-180-2-2-treated SA75 was ~9 times lower than that of the untreated SA75 (2.00% vs. 18.50%), notably, survival of the SYG-180-2-2-treated Newman was 850 times lower than that of the untreated Newman (0.02% vs. 17.00%; **Figure 4A**). Subsequently, we investigated whether SYG-180-2-2 sensitizes *S. aureus* to human whole blood. As shown in **Figure 4B**, the survival of SYG-180-2-2-treated Newman was ~7 times lower than that of the untreated Newman (1.50% vs. 10.00%), however, the survival of SYG-180-2-2-treated SA75 had no significance change compared with the untreated SA75 (2.75% vs. 4.25%). The human whole-blood killing assay suggested that a part of *S. aureus* was sensitive to human blood immune clearance after SYG-180-2-2 treatment.

### Effect of SYG-180-2-2 on the expression of genes involved in the virulence factors

**Figure 5:**
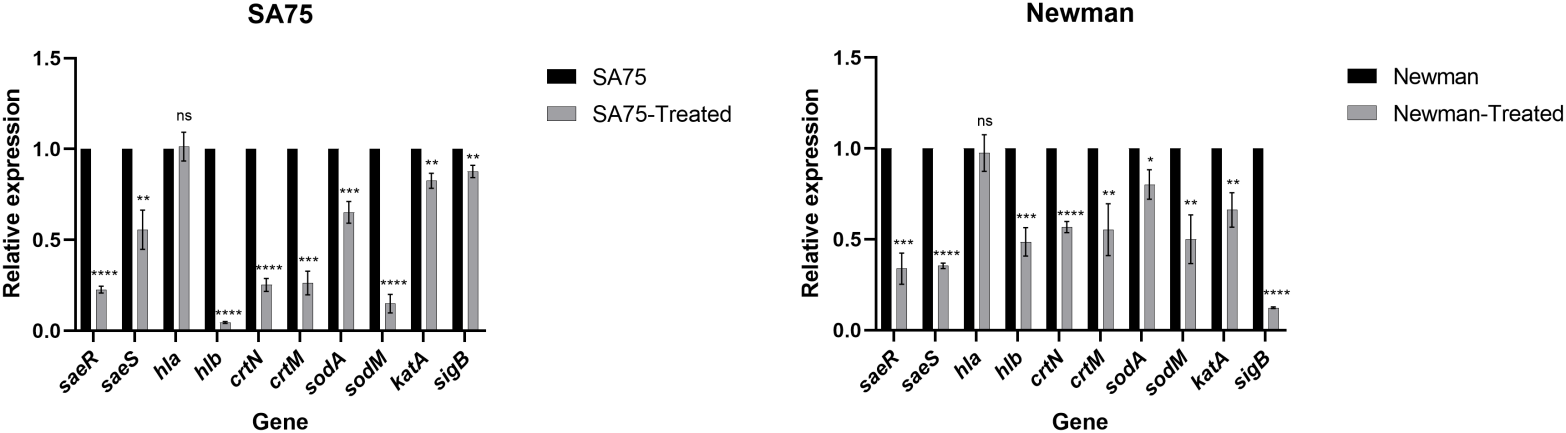
Expression changes of hemolysis and staphyloxanthin-associated genes in *S. aureus* upon SYG-180-2-2 treatment. ^*^ *P* < 0.05, ^**^ *P* < 0.01, ^***^ *P* < 0.001 and ^****^ *P* < 0.0001 and ns: not significant.

Through the hemolytic activity assay, we found that SYG-180-2-2 greatly reduced the hemolytic activity of *S. aureus*, so we analyzed the hemolysis-related genes *hla* and *hlb* from the transcriptional level. We found that there was no significance difference in the expression of *hla*, while the expression of *hlb* decreased by 21.77-fold in SA75 and 2.06-fold in Newman, respectively (**Figure 5**). We conducted RT-qPCR to explore the correlation between the expression of *hlb* and *saeRS*. We found that the expression of *saeRS* in strains treated with SYG-180-2-2 was significantly decreased (**Figure 5**). After SYG-180-2-2 treatment, the expression of *saeR* decreased 4.41-fold in SA75 and 2.95-fold in Newman, respectively. In order to further study the mechanism of SYG-180-2-2 on staphyloxanthin, we performed qPCR experiments of staphyloxanthin synthesis-associated genes and confirmed that the expression levels of *crtN*, *crtM* and *sigB* were significantly decreased by 3.96-fold, 3.80-fold and 1.14-fold in SA75 and 1.76-fold, 1.81-fold and 8.08-fold in Newman, respectively. (**Figure 5**). Further, qPCR confirmed that SYG-180-2-2 treatment also downregulated the expression of oxidative stress related genes such as *sodA*, *sodM* and *katA*, being 1.53-fold, 6.67-fold and 1.21-fold in SA75 and 1.25-fold, 2.00-fold and 1.60-fold in Newman, respectively (**Figure 5**).

### SYG-180-2-2 has no cytotoxicity to larvae and attenuates the virulence of *s. aureus*

**Figure 6:**
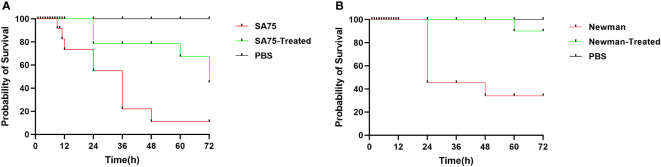
The survival rates of larvae after inoculated with 4 μg/mL SYG-180-2-2-treated or untreated SA75 **(A)** and Newman **(B)**. PBS was used as a negative control.

There was no death of larvae in the high-dose SYG-180-2-2 group (0.32 mg/kg) and the control group (inoculated with PBS) for 3 days, indicating that SYG-180-2-2 was not toxic within the tested dose. We found that the virulence of *S. aureus* treated with SYG-180-2-2 in larvae was significantly lower than that of the untreated *S. aureus*. After 72 hours, the death rates of SA75 and Newman treated with SYG-180-2-2 were reduced by 30% and 60%, respectively (**Figure 6**).

### SYG-18-2-2 has no injurious effect on the skin of mice

**Figure 7:**
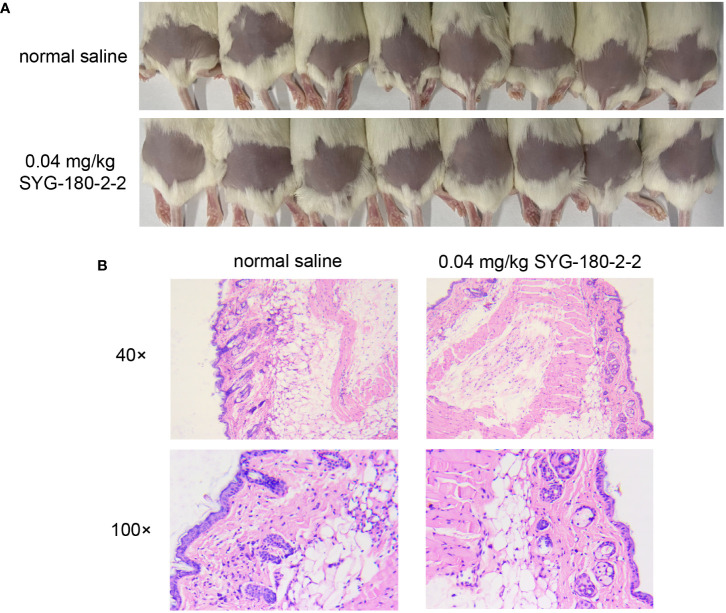
The safety evaluation of topical application SYG-180-2-2 *in vivo*. **(A)** Skin conditions of mice before and after SYG-180-2-2 treatment on day 4. **(B)** Pathological sections of representative mice between the SYG-180-2-2 group and the saline group.

As shown in **Figure 7A**, as in the control group, the skin of mice injected with SYG-180-2-2 was intact and there was no abscess formation. As depicted in **Figure 7B**, as in the control group, after three injections of 100 μL of 8 μg/mL SYG-180-2-2, the epidermis was intact, the dermis was not obvious damaged, and there was no obvious inflammatory cell infiltration. The result showed that SYG-180-2-2 had no damage to the skin of mice.

### SYG-180-2-2 is effective reduces the formation of skin abscess es in mouse after *s. aureus* infection

**Figure 8:**
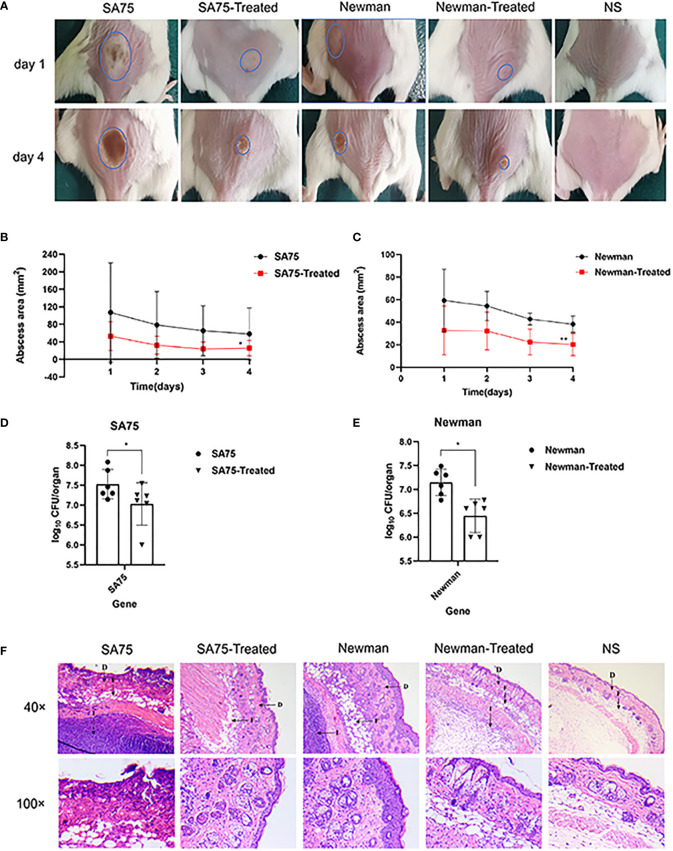
*In vivo* topical application of SYG-180-2-2 against *S. aureus*. **(A)** The image of representative abscesses before and after SYG-180-2-2 treated at day 1 and 4 after infection. **(B, C)** Daily changes of skin abscess area in mice before and after treatment. **(D, E)** Comparison of bacterial colonies in mice skin of *S. aureus* wild-type group and *S. aureus* treated group. **(F)** On day 4, pathological sections of representative mice between untreated group and treated group, containing two multiples (40× and 100×). D, Dermis; F, Fat layer; I, Inflammatory cells. ^*^ *P* < 0.05 and ^**^ *P* < 0.01.

We used a mouse skin abscess model to further explore the effects of SYG-180-2-2 on skin abscess formation and invasiveness of *S. aureus in vivo*. We injected the bacterial suspension subcutaneously into the back of mice, and the amount of injection was the same in each mouse. The sizes of abscess formed after infection were different, we tried to choose the same place and the area of abscess formation was easy to observe. As shown in **Figure 8A**, treatment with SYG-180-2-2 significantly restrained the size of abscess after 1 day of infection and significantly reduced the formation of yellow eschar on day 4 (blue circles). **Figures 8B, C** plotted changes in abscess area of SA75 and Newman before and after treatment with SYG-180-2-2, respectively. Abscesses in all groups were most pronounced on day 1 after infection, and then began to decrease and heal gradually. Notably, the abscess sizes of SA75 wild-type group were significantly larger than those of SA75-treated group on the first day post-infection (107.13 mm^2^ vs. 53.01 mm^2^). Mice were sacrificed 4 days after infection and bacterial load in skin was determined. After treatment, the loads of SA75 and Newman were decreased by ~0.7 log^10^ CFU/abscess and ~0.5 log^10^ CFU/abscess, respectively (**Figures 8D, E**). As depicted in **Figure 8F**, compared with the treated group, the skin sections of the untreated group showed more severe skin lesions and inflammatory cell infiltration. In particular, in the SA75-untreated group, the epidermis was completely destroyed, the dermis was severely damaged and immune cells were infiltrated, suggesting severe infection and deep inflammation. In the SA75-treated group, the epidermis was intact and the infiltration of subcutaneous immune cells was largely controlled. The Newman group also had a large skin recovery after treatment. These results showed that SYG-180-2-2 could weaken the virulence of *S. aureus*.

## Discussion

*Staphylococcus aureus* tends to cause skin and soft tissue infections and shows its pathogenicity by producing numerous virulence factors (Wen et al., 2018). The resistance of bacteria highlights the need to develop new antibacterial agents. Previously, we synthesized a small molecule compound SYG-180-2-2, which has a significant inhibitory effect on biofilm at 4 μg/mL. In this study, it was found that the new compound SYG-180-2-2 also had anti-virulence ability. It would be a boon if an antibacterial drug could not only reduce the formation of *S. aureus* biofilm but also attenuate the virulence of *S. aureus via* reducing the expression of multiple virulence factors. Therefore, we continued to investigate the anti-virulence ability of small molecule compound SYG-180-2-2, and then the mechanism was explored.

Inhibition of *S. aureus* toxins is a potential target for anti-virulence therapy (Clatworthy et al., 2007; Rasko and Sperandio, 2010; Kong et al., 2016). Hemolysin, in particular, is a crucial part of virulence. Alpha-toxin, a small pore-forming toxin, can cause skin and soft tissue infections (von Hoven et al., 2019). Beta-toxin, as the hot-cold hemolysin, is an important process occurring in infective endocarditis and sepsis (Herrera et al., 2016; Herrera et al., 2017). In our experiment, the hemolytic activity assay showed that 4 μg/ml SYG-180-2-2 could significantly reduce the hemolytic ability of *S. aureus*. At present, many FDA-approved drugs have been found to inhibit α-hemolysin, such as resveratrol, mupirocin and fusidic acid (Duan et al., 2018; Jin et al., 2018; Liu et al., 2020). However, we found no significance difference in *hla* expression and α-hemolysin release after SYG-180-2-2 treatment by transcriptional and protein level, namely RT- qPCR and ELISA kit quantitative α-toxin test. Meanwhile, real-time PCR showed that *hlb* gene expression decreased significantly with SYG-180-2-2-treated. Therefore, we speculate that SYG-180-2-2 may reduce the hemolytic ability of *S. aureus* by down-regulating the expression of *hlb* gene rather than *hla* gene. Intriguingly, we also observed that SYG-180-2-2 downregulated the expression of *saeS* and *saeR*. The *SaeRS* regulatory system plays an important role in the regulation of virulence gene expression in certain types of infections, including upregulating the transcription of *hla*, *hlb* and *coa* (Giraudo et al., 1999; Liang et al., 2006). SaeRS regulatory system was downregulated, which should lead to *hla* and *hlb* downregulation, however, we didn’t see significant change of the *hla* expression. The phenomenon may suggest other factors involved in *hla* regulations. We hypothesized that it might be related to the time when *saeRS* affected *hla* expression, or SYG-180-2-2 affected their transcription mechanism. We concluded that the downregulation of *saeR* gene might contribute to the downregulation of other virulence genes containing *hlb*, and weaken the virulence of *S. aureus*.

Targeting virulence factor staphyloxanthin is one of the alternative therapeutic strategies to control *S. aureus* infection (Xue et al., 2019; Ni et al., 2020; Elmesseri et al., 2022). In all experiments, cells treated with SYG-180-2-2 appeared white in color, which was significantly different from the golden yellow colored control cells. Based on this result, we then conducted studies on the efficacy of SYG-180-2-2 in inhibiting staphyloxanthin. Staphyloxanthin, a carotenoid pigment, has oxidative defense and protects *S. aureus* from host-mediated immune responses (Clauditz et al., 2006). Notably, staphyloxanthin was almost completely inhibited at the concentration of 4 μg/mL. As we all know, the production of staphyloxanthin was mediated by five enzymes, which was encoded by *crtOPQMN* operon (Pelz et al., 2005; Kim and Lee, 2012). In the biosynthesis of staphyloxanthin, when the cells appear white in color after treating, it is possible that drugs target *crtM*, *crtN* or other regulatory factors that affect the expression of the *crtOPQMN* operon, such as *sigB* (Gao et al., 2017; Pannu et al., 2019; Xue et al., 2019). In our study, RT-qPCR found that the transcription levels of *crtM* and *crtN* were downregulated after SYG-180-2-2 treatment, indicating the involvement of regulators. Interestingly, the expression of *sigB* had huge reduction in the SYG-180-2-2-treated *S. aureus* Newman. Therefore, we speculate that SYG-180-2-2 may inhibit the expression of *sigB*, thereby preventing the production of staphyloxanthin.

As staphyloxanthin has antioxidant activity, inhibition of this pigment will reduce the ROS resistance of *S. aureus* (Hall et al., 2017). In order to validate whether the decrease in pigment of *S. aureus* after SYG-180-2-2 treatment would affect its antioxidant ability, hydrogen peroxide killing experiment was performed. The results showed that the survival rate of *S. aureus* decreased significantly after treatment with SYG-180-2-2, confirming that the reduction of staphyloxanthin could make *S. aureus* more sensitive to ROS. Superoxide dismutases (SOD) is one of the major antioxidant enzymes that defend against ROS (Bhattacharyya et al., 2014). The decreased expression of the catalase gene *katA*, which protects bacteria from the damage caused by hydrogen peroxide, indicated that SYG-180-2-2 treatment increased the susceptibility of *S. aureus* to hydrogen peroxide. The decreased expression of SOD-related genes, *sodA*, *sodM* and *katA*, which protect bacteria form oxidative killing, further confirmed the increased sensitivity of *S. aureus* after SYG-180-2-2 treatment. Furthermore, the whole blood killing assay also verified that SYG-180-2-2 increased the sensitivity of part of *S. aureus* to healthy human blood by inhibiting staphyloxanthin production. Thus, staphyloxanthin is an important target for SYG-180-2-2 to reduce the virulence of *S. aureus*.

The absorption, metabolism and excretion mechanism of chemical substances in silkworm and mammals are similar (Hamamoto et al., 2005). Therefore, the use of silkworm infection model in preliminary drug screening can not only reduce the cost, but also more convenient to confirm whether drugs have toxicity and efficacy *in vivo* (Fujiyuki et al., 2010; Ishii et al., 2016). In our study, high dose SYG-180-2-2 injection of silkworm larvae *in vivo* had verified the preliminary safety of this compound. Additionally, the virulence of bacteria in larvae was significantly decreased after 4 μg/mL SYG-180-2-2 treatment. *S. aureus* usually causes skin infections, especially methicillin-resistant *S. aureus*, which is often accompanied by neutrophil migration and infiltration (Bassetti et al., 2017; Kwiecinski et al., 2021). The SYG-180-2-2 injection of mouse skin was further confirmed the safety of this compound. As shown in the mouse skin abscess model, we demonstrated the robust efficacy of SYG-180-2-2 against *S. aureus in vivo*. The skin abscess area, bacterial burden and inflammation were significantly improved by SYG-180-2-2. The reduced bacterial population of skin abscesses provided that reduced pigment in *S. aureus in vivo* may contribute to its decreased immune evasion ability. Notably, SYG-180-2-2 also had obvious curative effect on SA75 with larger skin infection area and more severe inflammatory infiltration.

Although SYG-180-2-2 has no toxicity in mouse skin and larvae, the safety of the compound in animals by intravenous injection and peros administrations have not been proven, and thus relevant trials are required in the future. We will evaluate the antibacterial effect of SYG-180-2-2 against *Staphylococcus aureus* with hemolysis and pigment-forming ability in the future. Meanwhile, further research is needed to detect the detailed mechanism of action of SYG-180-2-2. Overall, SYG-180-2-2 is a promising small molecule compound, and its multi-target anti-virulence in *S. aureus* may be more beneficial for the treatment of *S. aureus* infection.

## Data availability statement

The original contributions presented in the study are included in the article/**Supplementary Material**. Further inquiries can be directed to the corresponding authors.

## Ethics statement

The animal study was reviewed and approved by Ethics Committee of Shanghai Pulmonary Hospital, School of Medicine, Tongji University, Shanghai, China.

## Author contributions

LR, YX1, and LS designed the work and analyzed and interpreted the data for the work. LR and XW drafted the work and revised it critically for important intellectual content. ZS and FY provided approval for publication of the content. HZ, BW, JZ, YX2, YG, YS and LC participated in the experimental design and data analysis. ZS and FY agreed to be accountable for all aspects of the work in ensuring that questions related to the accuracy or integrity of any part of the work are appropriately investigated and resolved. All authors contributed to the article and approved the submitted version.

## Funding

This work was supported by the National Natural Science Foundation of China (81902122).

## Acknowledgments

We are grateful to colleagues in the pathology department of Shanghai Pulmonary Hospital, Tongji University School of Medicine for their assistance during preparation of pathological sections.

## Conflict of interest

The authors declare that the research was conducted in the absence of any commercial or financial relationships that could be construed as a potential conflict of interest.

## Publisher’s note

All claims expressed in this article are solely those of the authors and do not necessarily represent those of their affiliated organizations, or those of the publisher, the editors and the reviewers. Any product that may be evaluated in this article, or claim that may be made by its manufacturer, is not guaranteed or endorsed by the publisher.

## Supplementary material

Supplementary Figure 1 Growth curves of SA75 and Newman strains cultured with 8 μg/mL or without SYG-180-2-2. TSB was used as a blank control.

Supplementary Figure 2 Effect of SYG-180-2-2 on α-hemolysin was quantified by ELISA in SA75 and Newman strains grown with or without SYG-180-2-2. *P* > 0.05.
