# Supplementary figures and images for: Small-molecule compound SYG-180-2-2 attenuates Staphylococcus aureus virulence by inhibiting hemolysin and staphyloxanthin production
Source: Front Cell Infect Microbiol. 2022 Oct 13;12:1008289. doi: 10.3389/fcimb.2022.1008289 (PMC9606476; doi:10.3389/fcimb.2022.1008289)

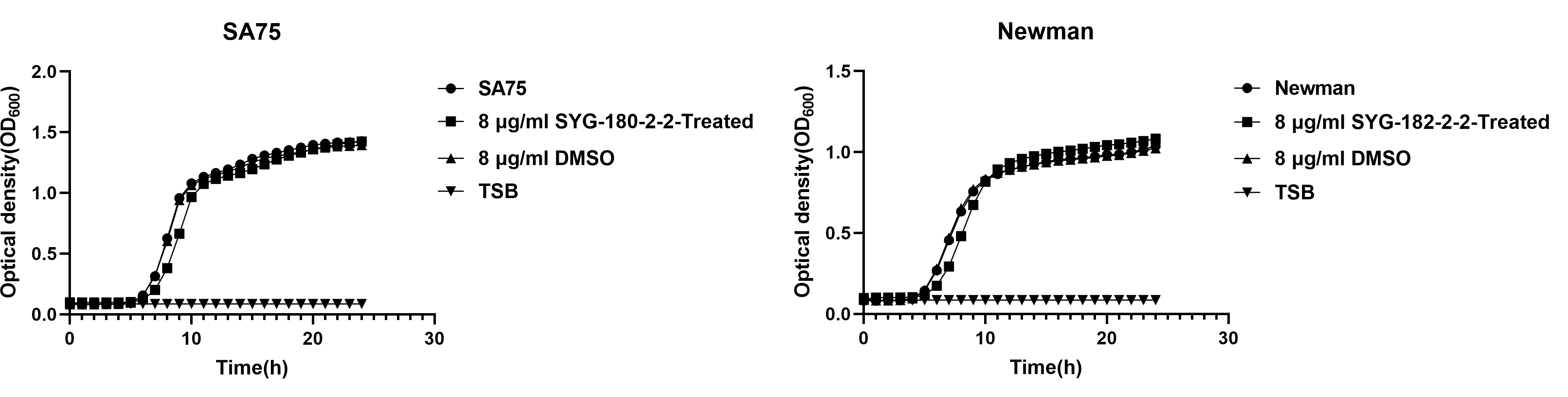

Supplement: Supplementary Figure 1 — Growth curves of SA75 and Newman strains cultured with 8 μg/mL or without SYG-180-2-2. TSB was used as a blank control. [file Image_1.tif]

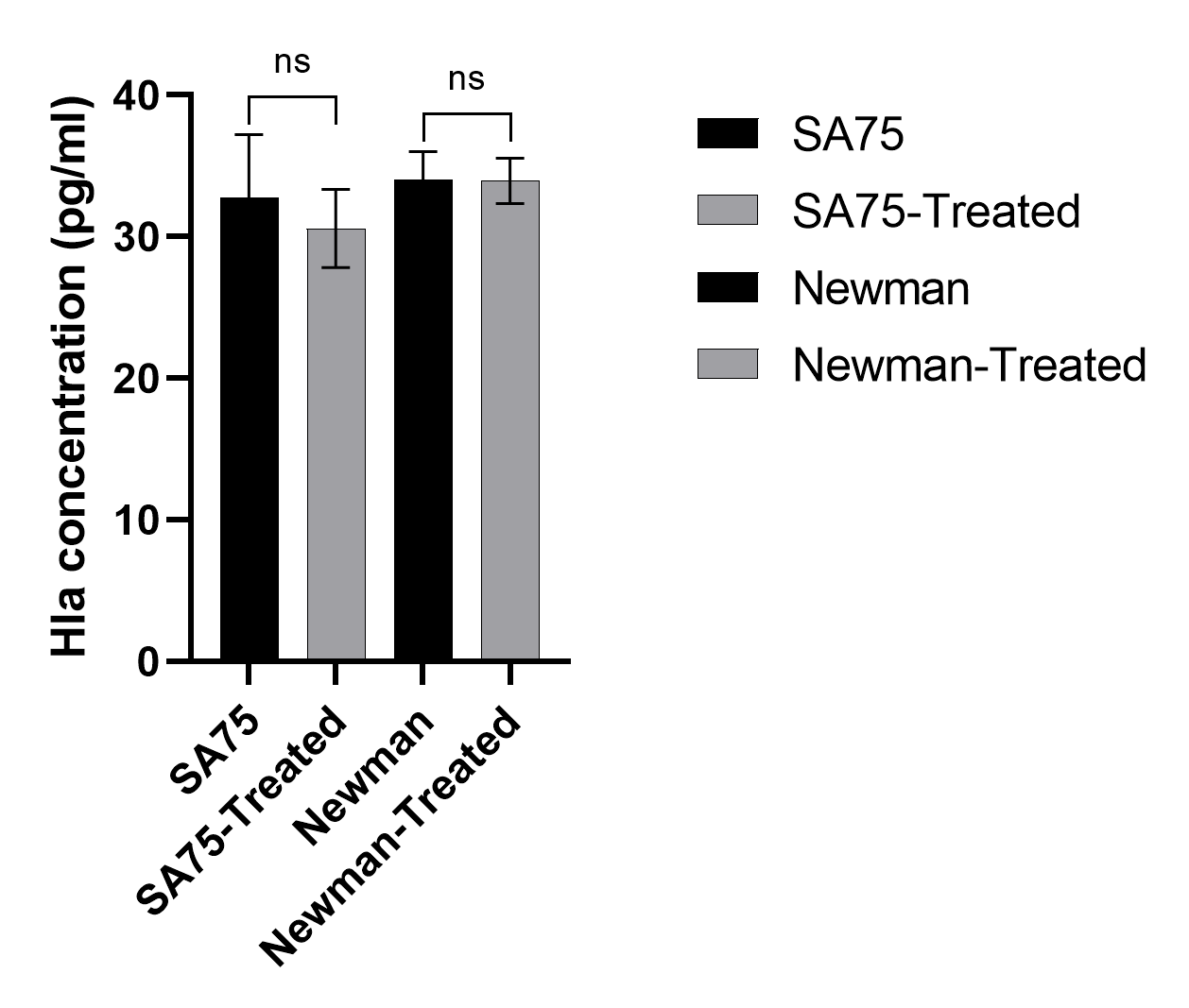

Supplement: Supplementary Figure 2 — Effect of SYG-180-2-2 on α-hemolysin was quantified by ELISA in SA75 and Newman strains grown with or without SYG-180-2-2. P > 0.05. [file Image_2.tif]
